# A Rare Case of p190 BCR-ABL Chronic Myeloid Leukemia With a Very Good Response to Tyrosine Kinase Inhibitors

**DOI:** 10.7759/cureus.16914

**Published:** 2021-08-05

**Authors:** Nalinikumari Gandhe, Mona Vekaria, Vrushali Dabak

**Affiliations:** 1 Internal Medicine, Henry Ford Health System, Detroit, USA; 2 Hematology and Medical Oncology, Henry Ford Health System, Wyandotte, USA; 3 Hematology and Medical Oncology, Henry Ford Health System, Detroit, USA

**Keywords:** chronic myeloid leukemia (cml), tyrosine kinase receptor inhibitors, dasatinib, p190 bcr-abl, nilotinib

## Abstract

The oncoprotein BCR-ABL has distinct fusion proteins generated from the Philadelphia chromosome translocation, depending on the site of the breakpoint on chromosome 22. The p210 is the hallmark of chronic myeloid leukemia. Only 1% - 2% of patients with chronic myeloid leukemia (CML) demonstrate p190 BCR-ABL. Imatinib mesylate, a tyrosine kinase inhibitor (TKI), specifically targets BCR-ABL, which brought a revolutionary era to the treatment of CML. Although the efficacy of imatinib is widely known, resistance to it has become a pressing challenge in the treatment of CML. CML patients harboring atypical e1a2 transcript (referred to as p190 BCR-ABL) show a poor and short-lived response to first-generation TKI therapy. Patients with p190 BCR-ABL CML should be identified as high-risk patients from the beginning to allow the best chance of a deep molecular response. These patients must be closely monitored during TKI therapy and should be treated upfront with a second-generation TKI. We report a case of p190 BCR-ABL CML with a good response to second-generation TKI.

## Introduction

The oncoprotein BCR-ABL has distinct fusion proteins generated from the Philadelphia (Ph) chromosome translocation, depending on the site of the breakpoint on chromosome 22. The p210 is the hallmark of chronic myeloid leukemia (CML) [1-3]. The majority of patients with Philadelphia-positive acute lymphoblastic leukemia (Ph+ALL) [1] and only 1% - 2% of patients with chronic myeloid leukemia demonstrate p190 BCR-ABL [4-6].The most common BCR-ABL transcripts in CML are e13a2(b2a2) and e14a2(b3a2) [4] .Other transcripts, such as e1a2 (referred to as p190 BCR-ABL), are rare [4, 7]. In the most recent literature, p190 BCR-ABL CML seems associated with a poor outcome [5].

## Case presentation

**Figure 1:**
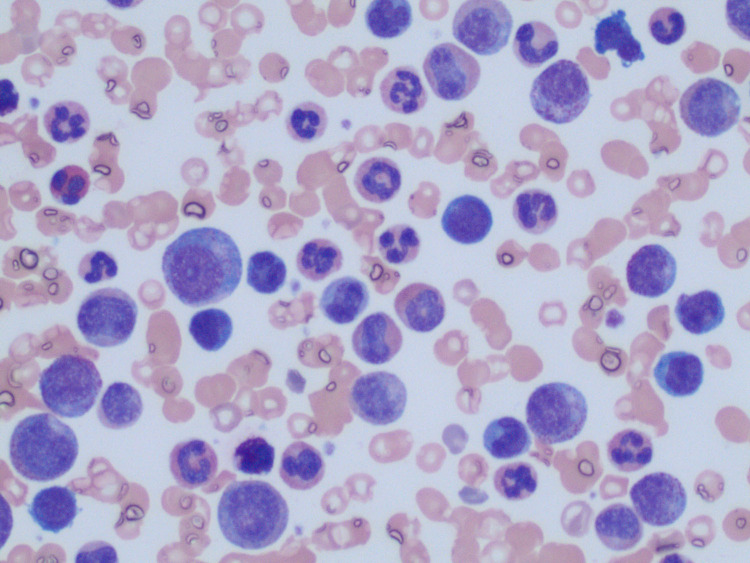
Peripheral blood smear Peripheral blood smear showing marked leukocytosis with left-shifted neutrophilia, myelocyte bulge, basophilia, and 1% blasts. There was moderate normocytic anemia with anisocytosis, polychromasia, and rare circulating nucleated red blood cells (RBCs).

We present the case of an 83-year-old female with a history of coronary artery disease status-post stent placement, hyperlipidemia, hypertension, and diabetes with no history of smoking who presented with worsening fatigue, night sweats, loss of appetite, and 15-pound weight loss. Physical examination was unremarkable, except for mild pedal edema. Laboratory studies were done with complete blood count and differential showing leukocytosis with a white blood cell count (WBC) of 303.5 K/uL (reference range: 3.8 - 10.6 K/uL) and anemia with a hemoglobin of 8.9 g/dL (reference range: 12 - 15 g/dL). Peripheral blood smear showed marked leukocytosis with left-shifted neutrophilia, myelocyte bulge, basophilia, and 1% blasts (Figure 1 ).

**Figure 2:**
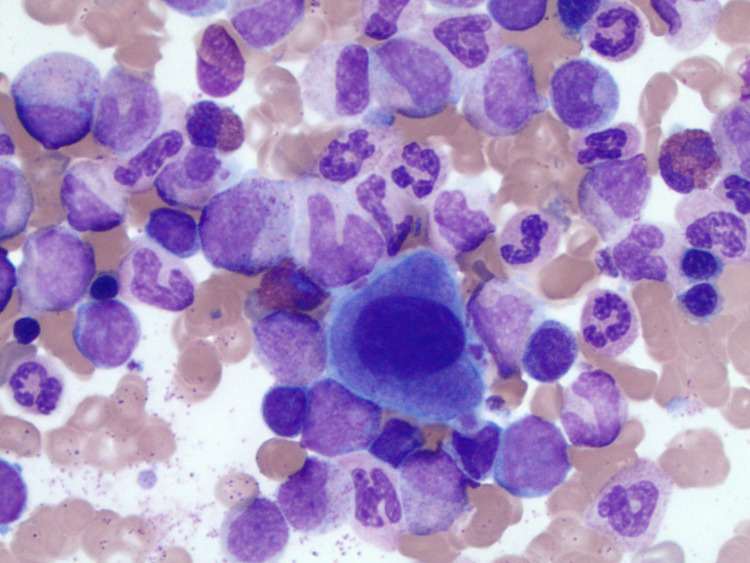
Bone marrow biopsy Bone marrow aspirate smear and core biopsy showing chronic myeloid leukemia (CML), BCR-ABL1-positive, chronic phase.

Peripheral blood cytogenetics fluorescence in situ hybridization (FISH) sent for t (9;22) was positive, confirming a CML diagnosis. However, molecular testing for BCR-ABL transcript was negative for p210 protein. Hence, given the clinical diagnosis was most consistent with CML, molecular reverse transcription-polymerase chain reaction (RT-PCR) for BCR-ABL p190 protein was checked. It showed that the ratio of BCR-ABL p190 transcripts to control gene transcript was 1.0775. Other laboratory studies showed an elevated uric acid which was concerning for pending tumor lysis syndrome. Hence, in the interim while the diagnosis was being confirmed, the patient was cytoreduced on hydroxyurea, 500 mg daily, and allopurinol, 100 mg daily. After two weeks, the WBC count was 107 K/uL (reference range: 3.8 - 10.6 K/uL), improving her fatigue and night sweats. A bone marrow biopsy showed CML, BCR-ABL-positive, chronic phase (Figure 2).

**Figure 3:**
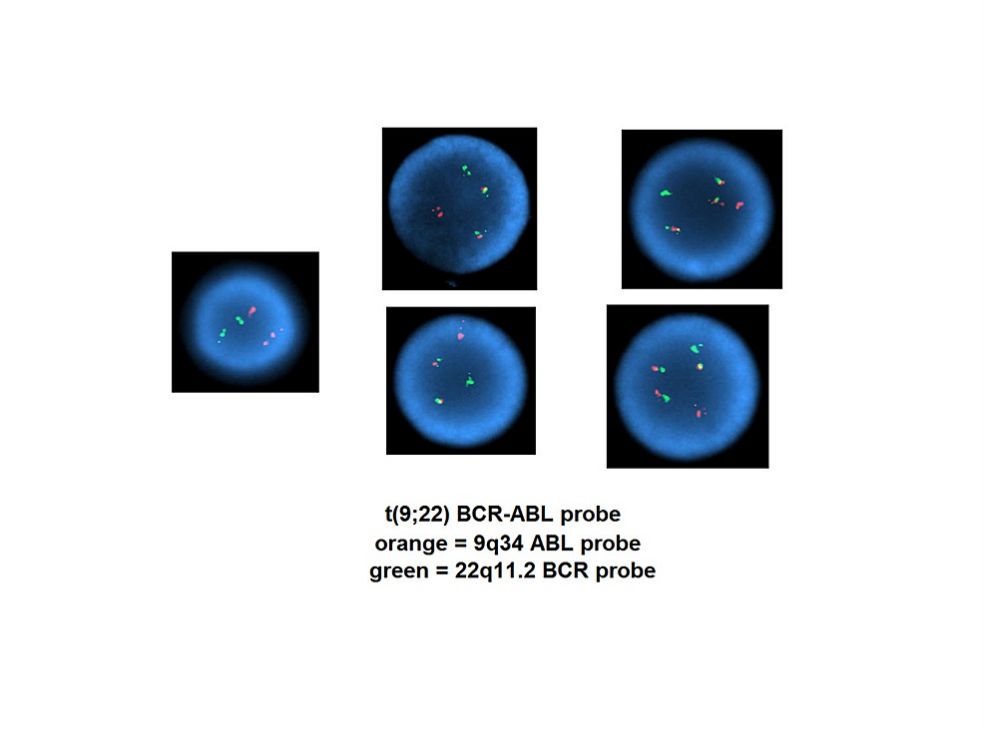
Cytogenetics fluorescence in situ hybridization (FISH) Chromosome analysis shows a t (9;22) translocation, which results in the fusion of the ABL1 gene a9q34 and the BCR gene at 22q11, as well as the gain of a derived chromosome 22 resulting from a t (9;22). The t (9;22) is consistent with a diagnosis of chronic myeloid leukemia (CML).

Cytogenetics FISH showed a t (9;22) translocation, which results in the fusion of the ABL1 gene at 9q34 and the BCR gene at 22q11, as well as the gain of a derived chromosome 22 resulting from a t (9;22) (Figure 3).

The t (9;22) was consistent with a diagnosis of CML. The gain of a second Ph chromosome is commonly seen in association with the impending blast crisis of CML, concerning for clonal evolution. However, this patient did not have clinical or hematopathological features of the impending blast crisis. The patient was started on dasatinib, 80 mg daily, instead of the standard 100 mg dose, given the high concerns for thrombocytopenia and worsening anemia.

After two months of treatment, repeated complete blood count and differential showed anemia and thrombocytopenia, concerning for chemotherapy-induced toxicity. Hence, the dasatinib dose was reduced to 70 mg daily. At this point, repeat molecular RT-PCR showed that the ratio of BCR/ABL p190 transcripts to control gene transcripts was 0.1645, consistent with disease response. FISH showed t (9;22) to BCR-ABL fusion, as well as separate t (9;22) BCR-ABL fusion, with a gain of derived chromosome 22 indicating the persistence of the abnormal clone found at diagnosis. 

At four months post-diagnosis, she had a hospitalization after a fall. Computerized tomography (CT) of the chest showed a large left pleural effusion that required thoracentesis. Cytology of the pleural fluid was negative. The pleural effusion was ultimately felt to be secondary to complications of dasatinib. Hence, the medication was switched to nilotinib, 200 mg twice daily. At that time, the molecular RT-PCR showed that the ratio of BCR/ABL p190 transcripts to control gene transcripts was 0.0766, consistent with disease response. FISH reported t (9;22) to BCR-ABL1 fusion, and the derived clonal population showed a partial response. The patient’s anemia and thrombocytopenia remained stable. The nilotinib dose was increased to 400 mg in the morning and 200 mg in the evening. At eight months after the diagnosis, the molecular RT-PCR showed that the ratio of BCR/ABL p190 transcripts to control gene transcripts was 0.0147 and FISH showed essentially near-complete response. To date, almost 11 months since diagnosis, our patient’s most recent molecular RT-PCR showed the ratio of BCR/ABL p190 transcripts to control gene transcripts was 0.0048.

## Discussion

**Figure 4:**
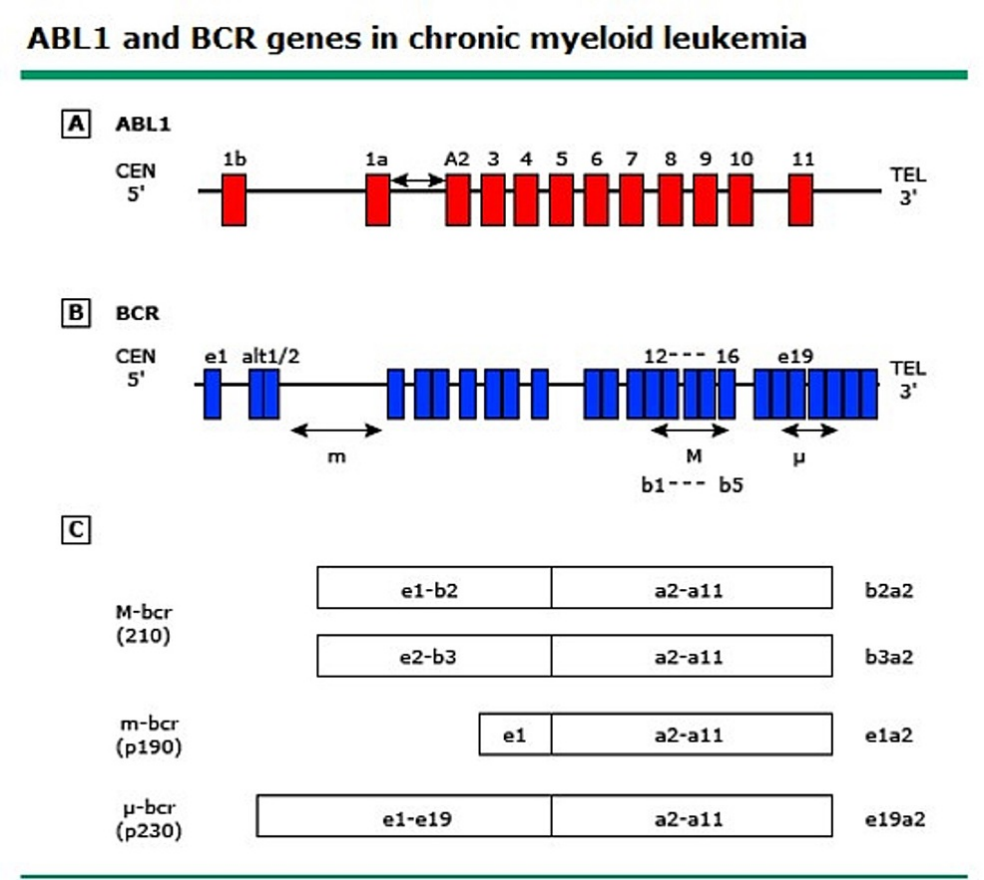
ABL and BCR genes in chronic myeloid leukemia.

About 95% of chronic myeloid leukemia (CML) cases present at diagnosis with the t (9;22) (q34.1; q11.2) translocation, which fuses sequences of the BCR gene on chromosome 22 with regions of ABL1 on chromosome 9. The resulting fusion protein may be different in size, based on the breakpoint in the BCR gene. Major p210, minor p190, and micro p230 variants are the resulting breakpoint cluster regions [5, 8]. The breakpoint in the BCR gene on chromosome 22 usually occurs in the major breakpoint cluster region (M-BCR) between exons e12-e16 (formerly named b1-b5), whereas breakpoints in the ABL gene on chromosome 9 happen in exon a2, resulting in fusion transcripts e13a2(b2a2) and e14a2(b3a2) [4, 9]. Rarely, the minor breakpoint cluster region may be involved with a resultant fusion transcript e1a2 [4]. The e13a2(b2a2)/e14a2(b3a2) fusion transcripts encode for a 210-kDa protein (p210 BCR-ABL), whereas the e1a2 encodes for a 190-kDa protein (p190 BCR-ABL) [9] and the e19a2 encodes for a 230-kDa protein (p230 BCR-ABL) [4] (Figure 4 ).

In CML, the e1a2 transcripts may coexist with e13a2(b2a2)/e14a2(b3a2). However, CML expressing only e1a2 transcripts (referred to as p190 BCR-ABL) is uncommon and is associated with an inferior outcome to the standard of care management with tyrosine kinase inhibitors. These patients tend to have short-lived responses [10].

Minor p190 BCR-ABL translocation is rare and only occurs in 1% - 2% of CML patients [5-6]. As reported by various authors, p190 BCR-ABL CML is often associated with peripheral monocytosis, absence of splenomegaly, and bone marrow morphologic features that are intermediate between CML and chronic myelomonocytic leukemia (CMML) [5, 7]. In our patient, while she had monocytosis that responded to therapy, she did not have splenomegaly or bone marrow features suggestive of transformation. She was intentionally started on high-risk management with second-generation tyrosine kinase inhibitor (TKI) therapy, but her comorbidities and complications proved to be a challenge and limitation during her treatment course. Despite this, she continues to have an adequate response. We would anticipate close to deep molecular response and complete cytogenetic response at 12 months, which would increase her ability to maintain the response in the future.

## Conclusions

p190 BCR-ABL translocation is rare and only occurs in 1% - 2% of chronic myeloid leukemia patients. Patients with p190 BCR-ABL CML show a poor and short-lived response to first-generation TKI therapy. Therefore, it is important to identify these patients as high-risk from the beginning to allow the best chance of a deep molecular response. Patients with p190 BCR-ABL CML should be treated upfront with second-generation TKI to achieve milestones in a timely fashion.
